# Inoculation of Barley (*Hordeum vulgare*) with the Endophyte *Epichloë bromicola* Affects Plant Growth, and the Microbial Community in Roots and Rhizosphere Soil

**DOI:** 10.3390/jof8020172

**Published:** 2022-02-10

**Authors:** Jing Liu, Zhengfeng Wang, Zhenjiang Chen, James F. White, Kamran Malik, Taixiang Chen, Chunjie Li

**Affiliations:** 1 State Key Laboratory of Grassland Agro-Ecosystems, Key Laboratory of Grassland Livestock Industry Innovation, Ministry of Agriculture and Rural Affairs, Engineering Research Center of Grassland Industry (Ministry of Education), Gansu Tech Innovation Centre of Western China Grassland Industry, Centre for Grassland Microbiome, College of Pastoral Agriculture Science and Technology, Lanzhou University, Lanzhou 730020, China; liuj16@lzu.edu.cn (J.L.); chenzj@lzu.edu.cn (Z.C.); malik@lzu.edu.cn (K.M.); chentx@lzu.edu.cn (T.C.); 2 Economic Crops and Malt Barley Research Institute, Gansu Academy of Agricultural Science, Lanzhou 730070, China; wangzhf1006@163.com; 3 Department of Plant Biology, Rutgers University, New Brunswick, NJ 08901, USA; jwhite3728@gmail.com; 4 Grassland Research Center of National Forestry and Grassland Administration, Chinese Academy of Forestry Sciences, Beijing 100091, China

**Keywords:** *Epichloë bromicola*, novel symbionts, peramine, nutritional properties, 16s rRNA, ITS rDNA

## Abstract

*Hordeum vulgare* is an important source of feed and forage for livestock, and of food and drink for humans, but its utilization rate is lower than that of other cereal crops, thus it is crucial to improve barley agronomic traits and production. *Epichloë bromicola* is an endophyte that was isolated from wild barley (*Hordeum brevisubulatum*). Previous studies have found that *Epichloë* can indirectly influence the growth of host plants by affecting soil chemical characteristics, the microbial community, and by producing a range of secondary metabolites. However, underlying effects of *Epichloë* on the abundance and diversity of soil and root microbes have not been well-studied. In addition, there is a question regarding the relationship between endophyte-produced alkaloids and effects on the root and rhizosphere microbial communities. The objective of this study was to investigate changes in agronomic traits, nutritional properties, peramine, soil chemical and microbial community in the fourth generation of new barley symbionts EI (*E. bromicola*-infection) and EF (*E. bromicola*-free) in LQ+4 and LZ+4. We understand the plant height and biomass of EI in LZ+4 were significantly higher than those of EF. The HPLC analysis showed that the peramine content of EI in LQ+4 and LZ+4 was 0.085 and 0.1 mg/g, respectively. We compared the bacterial and fungal communities by analyzing the 16s rRNA (for bacteria) and ITS rDNA regions (for fungi). Our data revealed that the composition of fungal communities in rhizosphere soil of LZ+4 EI are higher than EF. In addition, the diversity and richness of fungal communities in root and rhizosphere soil of LQ+4 EI and LZ+4 EI are significantly higher than EF. Rhizosphere soil microbial community composition was higher than that in roots in LQ+4 and LZ+4. Peramine was significantly and positively correlated with the richness of the soil fungal community. Moreover, the principal component analysis (PCoA) results indicated that *E. bromicola* significantly influenced the community composition of root and rhizosphere soil microbes in both LQ+4 and LZ+4. Our results illustrate that *E. bromicola* can influence barley growth, peramine production and microbial communities associated with barley.

## 1. Introduction

*Epichloë* are common and diverse microorganisms which systemically colonize the intercellular spaces of leaf primordia, leaf sheaths and blades of tillers, and the inflorescence tissues of reproductive tillers [1]. Asexual symbionts are only vertically transmitted via the mother plant lineage [2]. Extensive studies have confirmed that *Epichloë* form symbioses with temperate grasses in the Pooideae subfamily, which enhanced resistance to biotic and abiotic stresses including drought, waterlogging, salt, cold, heat, heavy metals, insects, nematodes and diseases [2,3,4]. Moreover, *Epichloë* can indirectly affects the growth of the host plant by altering the host cell structure and secreting auxin and effector molecules into host cells or apoplasts [5,6]. *Epichloë*-grass symbiosis produce a range of alkaloids, which protect the plant from herbivores [7] in exchange for a protected niche and nutrition from the host plant [8,9].

Host plants inhabited by *Epichloë* are unique models for studying the potential relationship between aboveground and underground microbial communities [10]. There is growing evidence that *Epichloë* presence aboveground can have positive, neutral or negative effects belowground. For example, the endophyte-infection of tall fescue increased the OTUs of beneficial bacteria Proteobacteria and Acidobacteria [10], but it decreased the abundances of Gram-positive bacteria, arbuscular mycorrhizae [11], or showed no change in the community composition [12]. Other studies report that endophyte-infection of tall fescue have lower soil microbial activity [13]. In addition, *Epichloë* may provide a competitive advantage for hosts by affecting soil microbial processes and soil microbial communities [14]. In turn, soil bacterial and fungal community composition may regulate the plant endophytic diversity and community composition [15]. Plant roots release various molecules into the rhizosphere, thereby changing soil chemistry and providing a source of nutrients that can be used by resident microbes [16]. Some studies showed that alkaloids, as secondary metabolites of the host, may also affect the host microbial community [17,18], However, other researchers oppose this view [19,20]. Moreover, there are few studies that evaluate whether peramine produced by *Epichloë* may affect the microbial communities in roots or soils.

Barley (*Hordeum vulgare*) is distributed worldwide and ranks fourth in global cereal production [21]. Cultivated barley mainly includes *H. vulgare* cv and *H. vulgare* var. nudum cv, which are important for livestock feed and forage, as well as human food and drink, respectively [22]. Germplasm resources for barley are thus important for improvement of cultivars [23]. The use of endophytic fungi to improve the quality of forage and food cultivars offers a potential way to improve [24]. At present, the use of *Epichloë* for breeding in grasses is only used for some turfgrass and a few forages [25]. For example, novel endophyte AR542 and AR584 have been inserted into tall fescue (*Festuca arundinacea*) cultivar “Jesup” and “Texoma” and commercialized as “Jesup” MaxQ and “Texoma” MaxQ II, respectively [26]. The AR601 associated with the tall fescue cultivar ‘Jackal’ was used to develop the variety known as Avanex [27]. The endophytic fungal strains AR1 and AR37 were successfully inoculated into tall fescue and perennial ryegrass (*Lolium perenne*) through artificial inoculation methods to obtain varieties that were commoditized in the U.S. and Australia [28]. The main fungal endophyte inoculation methods for grasses include coleoptile inoculation, sterile seedling meristem inoculation, wound inoculations method, callus inoculation and the injection method [29,30,31,32]. However, the application of endophytic fungi to improve barley has not been reported previously.

Our team used *Epichloë bromicola* isolated from wild barley (Hordeum brevisubulatum) to artificially inoculate two varieties of cultivated barley LQ (Chaiqing No. 1) and LZ (Yangsimai No. 1), creating new germplasm of barley containing *E. bromicola*. Li [33] preliminarily found that the new barley germplasm increases aboveground biomass and seed yield. Studies on the effects of *E. bromicola* on the plant growth parameters, nutritional quality, peramine, soil chemical properties, and root and rhizosphere soil microbial communities of new barley symbionts have not been reported. Our objective was to evaluate the effect *Epichloë* interaction with barley on plant growth and microbial communities in the root and rhizosphere soil. Based on previous findings, three questions were considered in this study. The first question is whether *E. bromicola* affects barley growth, peramine production and nutritional quality of LQ and LZ. The second question is whether *E. bromicola* negatively affects the root and rhizosphere soil microbial communities. The third question is whether peramine produced by the *Epichloë* affects microbial communities in root and rhizosphere soils.

## 2. Materials and Methods

### 2.1. The Origin of Seeds

LZ (*H. vulgare* cv. Yangsimai No. 1) was purchased from Jiangsu Suqian Dijing Landscaping Engineering Co., Ltd. in Suqian, China. LQ (*H. vulgare* var. nudum cv. Chaiqing No. 1) was a new highland barley variety bred by Qinghai Linong Seed Industry Co., Ltd., Haixi Prefecture Seed Management Station and Qinghai Provincial Seed Management Station in Haixi, China. *E. bromicola* strain wbe1, isolated from wild barley, was provided by the college of Pastoral Agriculture Science and Technology, Lanzhou University, Lanzhou, China [34]. LZ and LQ were not infected with *Epichloë bromicola*. To inoculate barley, we placed endophyte mycelium into coleoptile tissue of axenic seedlings growing on water agar in Petri dishes. The first generation of barley (LZ+1, LQ+1) came from aseptic seedlings inoculated planted in a greenhouse, in 2017 [35]. The second (LZ+2, LQ+2), third (LZ+3, LQ+3), and fourth (LZ+4, LQ+4) generation of *E. bromicola*-infection barley was planted in Baiyin county experimental station of Lanzhou University in Chinaon March, in 2018, 2019, and 2020, respectively. We used the aniline blue staining method to determine the presence of *Epichloë* in the fourth (LZ+4, LQ+4) generation barley.

### 2.2. Site Description

The experimental site at Jingyuan county experimental farm (elevation of 1400 m) of the Baiyin Institute of Agricultural Sciences, Gansu Province, China, was used from March 2020 to August 2020. The experiment plots of EI (fourth generation) and EF were laid in a completely random design. The EI and EF seeds were sowed on 3 m × 4 m plots, using a 30 cm row spacing and plant spacing within rows, respectively. Each pot had four rows, each row length is one meter, four replicates, totaling 16 plots.

### 2.3. Field-Data Acquisition

After the plants were mature, we randomly selected 10 plants from each plot. The plant heights and stem thicknesses were determined. The grain numbers per spike and tillering per plant were determined. Biomass, yield per plant and thousand-grain weight were measured determined.

### 2.4. Plant Material

Plants were cut 5 cm above the soil, and fresh weights determined. Shoot tissues were dried (75 °C) to a constant weight for determining plant biomass. The residual aboveground parts and all roots were carefully removed from the soil, and thoroughly washed with distilled water before manual separation into the root and stem parts. The roots and stems were separately collected and placed in an icebox and transported to the laboratory. The stem samples were stored at −80 °C before peramine extraction. The root samples were gently washed with tap water several times then rinsed with sterile water, and then were dried with sterile filter paper. These root samples were stored at −80 °C prior to DNA extraction.

### 2.5. Peramine Extraction and Detection

The freeze-dried material (20 mg) was extracted and analyzed for peramine. Peramine was extracted according to the methods of Lin [36].

Alkaloids were quantified on an Agilent 1100 HPLC, following a protocol adapted from Lin [36]. The high-performance liquid chromatograph was an Agilent 1100 fitted with a C18 column (Eclipse XDB-C18, 250 mm × 4.6 mm, 5 μm). Detection was performed with an ultraviolet (UV) wavelength spectrophotometric detector set at 280 nm. The mobile phase consisted of solution A (1.8 g L^−1^ guanidine carbonate), which adjust pH to 3.7 with formic acid and solution B (acetonitrile). All reagents were chromatographically pure. An amount of 20 μL of extracted sample was injected into the injection port. Peramine alkaloid concentration was quantified using external standard curves.

Standard samples of peramine alkaloids were provided by Wade Mace, (AgResearch, Palmerston North, New Zealand).

### 2.6. Soil Sampling

Rhizosphere soil and bulk soil were collected in August 2020 at the conclusion of the seed harvest. For each subplot, four bulk soil samples, 15 cm deep, were taken from four individual barley plants using a 20 cm soil auger, four rhizosphere soil samples were taken from the residual soil remaining on the root, and then these samples were mixed to form a composite sample, respectively. Rhizosphere soil samples were placed in an icebox and transported to the laboratory and stored at −80 °C prior to DNA extraction. Bulk soil samples were passed through a 2.0 mm sieve and homogenized, then stored at 4 °C before soil chemical analysis.

### 2.7. Soil Chemical Analysis

The extraction of total phosphorus (TP) and total nitrogen (TN) in soil was digested with H2SO4 under catalyzed condition (CuSO4:K2SO4: 1:10 mixture) on a digestion block at 420 °C for 2 h and 1 h, respectively. The concentrations of TN and TP were determined by a continuous flow analyzer (FIAstar 5000 Analyzer, Foss, Denmark) [37]. To analyze Organic carbon (OC) content in soil, each sample was oxidized by K2CrO7-H2SO4 at 180 °C for 5 min, followed by titration with FeSO4 [38].

### 2.8. DNA Extraction, Nest PCR and Sequencing

For total DNA of root and soil samples, 0.2 g root and soil of each sample were extract DNA using NucleoSpin Soil Kit (Macherey-Nagel GmbH & Co. KG, Valencienner, Germany) following the manufacturer’s instructions. The DNA quantity was determined using a Qubit Fluorometer by using Qubit dsDNA BR Assay kit (Invitrogen, Waltham, MA, USA) and the quality was tested with running aliquots on 1% agarose gel. Extracts were stored at −20 °C.

For the bacterial sequencing library, we targeted the 16S rRNA V3–V4 gene region (341F: 5′-ACTCCTACGGGAGGCAGCAG-3′, 806R: 5′- GGACTACHVGGGTWTCTAAT-3). For the fungal sequencing library, we targeted the ITS1-ITS2 region (its1: 5′- CTTGGTCATTTAGAGGAAGTAA-3′; its2: 5′- GCTGCGTTCTTCATCGATGC -3′). PCR cycling conditions of universal primer-tailed 16S primers were as follows: 94 °C for 3 min, 30 cycles of 94 °C for 30 s, 56 °C for 45 s, 72 °C for 45 s and final extension for 10 min at 72 °C for 10 min. PCR cycling conditions of universal primer-tailed ITS primers were as follows: 94 °C for 3 min, 30 cycles of 94 °C for 30 s, 55 °C for 45 s, 72 °C for 45 s and final extension for 10 min at 72 °C for 10 min.

The PCR products were purified with AmpureXP beads and eluted in Elution buffer to qualify with the Agilent 2100 bioanalyzer (Agilent, USA. Amplicons were sent to the BGI for sequencing on the Illumina MiSeq platform (BGI, Shenzhen, China).

### 2.9. Sequencing and Bioinformatics Analysis

We filtered the raw reads to remove adaptors and low-quality and ambiguous bases, and we added paired-end reads to tags by the Fast Length Adjustment of Short reads program (FLASH, v1.2.11) [39]. We clustered tags into OTUs with a cutoff value of 97% using UPARSE software (v7.0.1090) [40] and detected the chimera sequences using UCHIME (v4.2.40) [41] compared with the UNITE (v20140703). We taxonomically classified sequences using Ribosomal Database Project (RDP) Classifier v.2.2 with a minimum confidence threshold of 0.6, and the sequences were trained on the UNITE (V6 20140910) by QIIME v1.8.0 [42]. We compared all Tags back with OUT by USEARCH global [43] to get the OTU abundance statistics table of each sample.

Alpha and beta diversity were estimated by MOTHUR (v1.31.2) [44] and QIIME (v1.8.0) [42] at the OTU level, respectively.

Shannonn (http://www.mothur.org/wiki/Shannon, accessed on 28 January 2022) and Simpson (http://www.mothur.org/wiki/S-impson, accessed on 28 January 2022) diversity indices were determined using R software (Version 2.15.3). It was proposed by Edward Hugh Simpson (1949) and often used in ecology to quantitatively describe the biodiversity of an area. Moreover, it can be employed to estimate the indices of microbial diversity in a sample [45]. Community diversity was determined with Shannon, and the calculation formula is as follows:

$$H_{shannon}=-\overset{S_{obs}}{\underset{i=1}{\sum }}\frac{n_{i}}{N}ln\frac{n_{i}}{N}$$

where the *S_obs_* represents the number of observed OTUs; *n_i_* is the sequence number contained in the OTUs; *N* is all of the sequence numbers. The higher Shannon value indicates higher community diversity.

Richness index Chao1 (http://www.mothur.org/wik-i/Chao, accessed on 28 January 2022) was determined using R software (Version 2.15.3). It is an index for estimating the number of OTUs present in a sample using the chao1 algorithm, which is used to calculate the total number of species in ecology. Chao1 index reflects community richness. It was first proposed by Chao (1984) [46]. The calculation formula is as follows:

$$chao1=S_{obs}+\frac{F_{1}{}^{2}}{2F_{2}}$$

where *S_obs_* represents the number of OTUs actually observed; *F*_1_ and *F*_2_ are the number of singletons and doubletons in each sample, respectively.

Principal component analysis (PCoA) of root and rhizosphere soil bacterial and fungal communities in LZ+4 and LQ+4 infected with EI and EF, based on phylum levels, were performed using Bray–Curtis dissimilarities by the R-package Vegan [42].

Redundancy analysis (RDA) of rhizosphere soil and root fungal and bacterial communities, soil properties and peramine in LZ+4 and LQ+4 infected with EI and EF, based on Spearman’s product-moment correlation and Bioenv function, were performed by CANOCO for Windows 4.5.

### 2.10. Statistical Analyses

Statistical analyses were performed using SPSS 22.0 (SPSS, Chicago, IL, USA) and Microsoft Office Excel 2010. Effect of endophyte status (EF and EI) and barley (LZ, LQ) on soil chemical property, barley quality, peramine and plant growth parameters were analyzed with a two-way ANOVA. Whether the differences between the means were statistically significant was assessed by Tukey’s-b (k) test at *P* = 0.05. Correlation analysis was employed to assess the relationships among *E. bromicola*, barley, soil chemical properties, nutritional quality, plant growth parameters, peramine, root and rhizosphere soil microbial community using Pearson’s method. In all tests, a *P* value < 0.05 was considered statistically significant.

## 3. Results

### 3.1. The Infection Rate by Endophytes

The average infection rate was observed by microscopic examination of the seeds and tillers. The average infection rate per plant in LZ tillers and seed were 76% and 56%, respectively, in 2020. The average infection rate per plant in LQ tillers and seeds were 68% and 47%, respectively, in 2020. The infection rate of LZ is greater than that of LQ (Table S1).

### 3.2. Barley Growth Parameters

**Figure 1:**
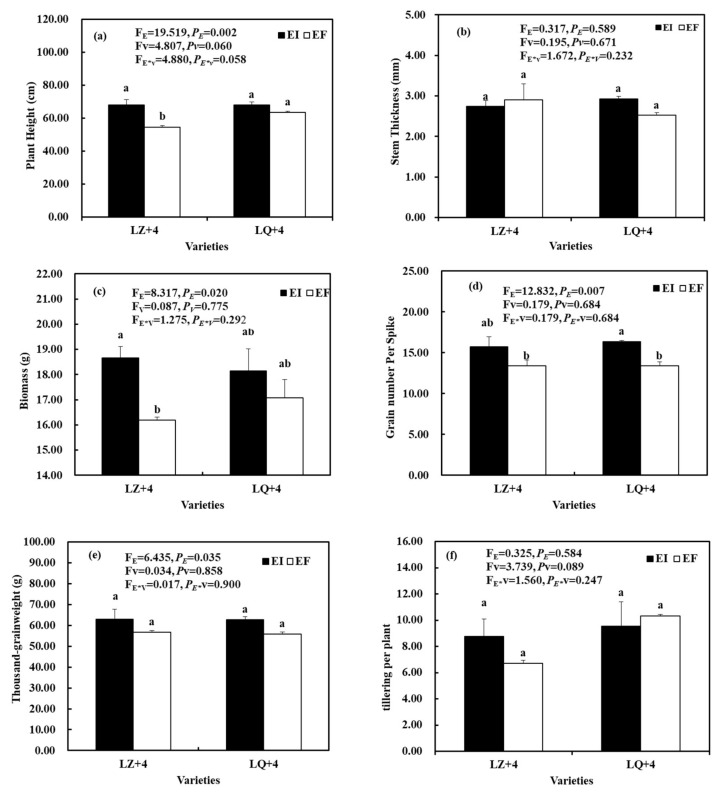
Plant growth parameters associated with barley (LZ and LQ) that were *Epichloë*-infection (EI) and *Epichloë*-free (EF). (**a**) plant height, (**b**) stem thickness, (**c**) biomass, (**d**) grain number per spike, (**e**) thousand-grainweight, (**f**) tillering per plant. Results are presented as mean ± SE. Different lowercase letters indicate significant differences (*P* < 0.05) between the EI and EF barley (*n* = 4, *P* < 0.05). *P_V_*-values, *P_E_*-values, and *P_VE_* of the ANOVA indicate significant differences *P* < 0.05 (independent *t*-test) endophyte status (E), varieties (V), and their interaction (E*V), respectively.

The *Epichloë* had a significant effect on plant height (F_E_ = 19.519, *P_E_* = 0.002) (Figure 1a), biomass (F_E_ = 8.317, *P_E_* = 0.020) (Figure 1c), grain number per spike (F_E_ = 12.832, *P_E_* = 0.007) (Figure 1d), and thousand-grain weight (F_E_ = 6.435, *P_E_* = 0.035) (Figure 1e), but the varieties and the interactions between the two factors were not significantly different (P_E__*V_ > 0.05) (Figure 1). The plant height and biomass were significantly higher in EI than in EF of LZ (Figure 1a,c). The grain number per spike was significantly higher in EI than in EF of LQ (Figure 1d). The *Epichloë*, varieties, and the interactions between the two factors have not significant different on stem thickness (Figure 1b) and tillering per plant (Figure 1f).

### 3.3. Peramine

**Figure 2:**
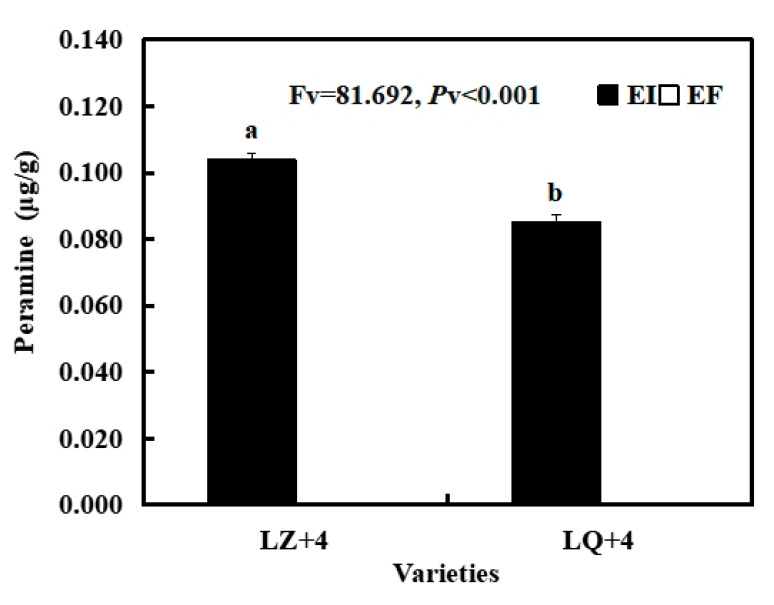
Peramine content of *Epichloë*-infection (EI) barley. Results are presented as mean ± SE. Different lowercase letters indicate significant differences (*P* < 0.05) (independent *t*-test) between the LZ and LQ barley with EI (*n* = 4, *P* < 0.05).

Peramine concentration was significantly higher in LZ with EI than in LQ with EI. There is no peramine in LZ with EF and LQ with EF (Figure 2).

### 3.4. Rhizosphere Soil Chemical Properties

Varieties had a significant effect on rhizosphere soil TP (F_V_ = 5.459, *P_V_* = 0.048) and N:P (F_V_ = 7.120, *P_V_* = 0.028), but the status of *Epichloë* (*P_E_* > 0.05) and the interactions between the two factors (P_E__*V_ > 0.05) were not significantly different (Table S2).

### 3.5. Composition of Microbial Community in Root and Rhizosphere Soil

#### 3.5.1. Composition of Fungal and Bacterial Community in Root and Rhizosphere Soil

**Figure 3:**
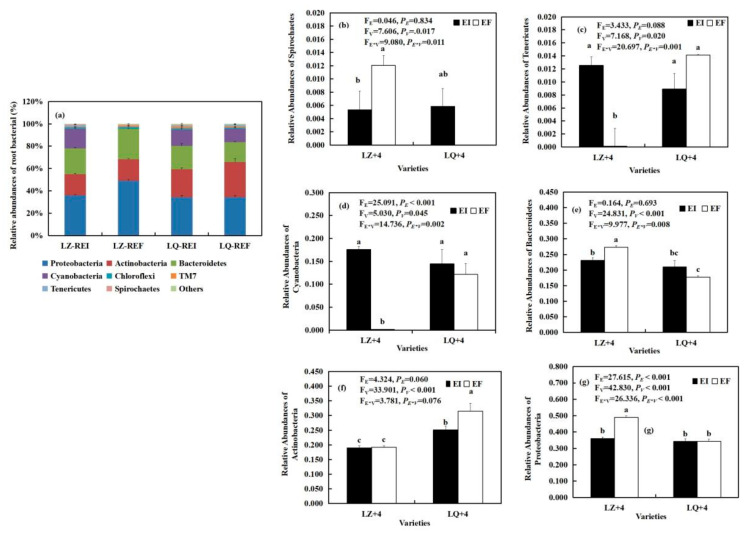
The relative abundance (at the phylum level) of root bacteria community found in *Epichloë*-infection (EI) and *Epichloë*-free (EF) plants of LZ+4 and LQ+4. REI: endophyte-infection root, REF: endophyte-free root. Results are presented as mean ± SE. (**a**) relative abundances of root bacterial, (**b**) relative abundances of Spirochaetes, (**c**) relative abundances of Tenericutes, (**d**) relative abundances of Cyanobacteria, (**e**) relative abundances of Bacteroidetes, (**f**) relative abundances of Actinobacteria, (**g**) relative abundances of Proteobacteria. Different lowercase letters indicate significant differences (*P* < 0.05) between the EI and EF barley (*n* = 4, *P* < 0.05). *P_V_*-values, *P_E_*-values, and *P_VE_* of the ANOVA indicate significant differences *P* < 0.05 (independent *t*-test) endophyte status (E), varieties (V), and their interaction (E*V), respectively.

**Figure 4:**
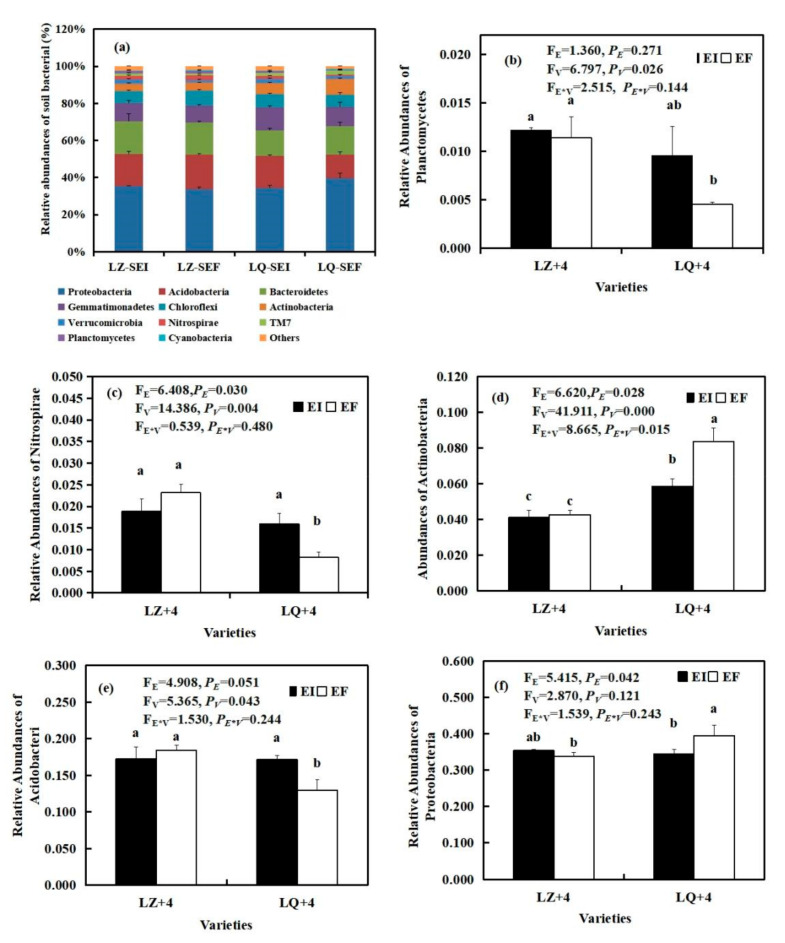
The relative abundance (at the phylum level) of rhizosphere soil bacteria community found in EI (*Epichloë*-infection) and EF (*Epichloë*-free) in LZ+4 and LQ+4. SEI: endophyte-infection rhizosphere soil, SEF: endophyte-free rhizosphere soil. Results are presented as mean ± SE. (**a**) relative abundances of soil bacterial, (**b**) relative abundances of Planctomycetes, (**c**) relative abundances of Nitrospirae, (**d**) relative abundances of Actinobacteria, (**e**) relative abundances of Acidobacteri, (**f**) relative abundances of Proteobacteria. Different lowercase letters indicate significant differences (*P* < 0.05) between the EI and EF barley (*n* = 4, *P* < 0.05). *P_V_*-values, *P_E_*-values, and *P_VE_* of the ANOVA indicate significant differences *P* < 0.05 (independent *t*-test) endophyte status (E), varieties (V), and their interaction (E*V), respectively.

By analyzing the main groups of root bacteria at the phylum level, we found that the *Epichloë* (E) significantly affected the relative abundances of Cyanobacteria (F_E_ = 25.091, *P_E_* < 0.001) (Figure 3d) and Proteobacteria (F_E_ = 27.615, *P_E_* < 0.001) (Figure 3g). Varieties (V) significantly affected the relative abundances of Actinobacteria (F_V_ = 33.901, *P_V_* < 0.001) (Figure 3f), Bacteroidetes (F_V_ = 24.831, *P_V_* < 0.001) (Figure 3e), Tenericutes (F_V_= 7.168, *P_V_* = 0.020) (Figure 3c), Spirochaetes (F_V_ = 7.606, *PV_S_* = 0.017) (Figure 3b), Cyanobacteria (F_V_ = 5.030, *P_V_* = 0.045) (Figure 3d) and Proteobacteria (F_V_ = 42.830, *P_V_* < 0.001) (Figure 3g). The *Epichloë* (E) and varieties (V) significantly affected the relative abundances of Bacteroidetes (F_E*__V_ = 9.977, P_E*__V_= 0.008) (Figure 3e), Tenericutes (F_E*__V_ = 20.697, P_E*__V_ < 0.001) (Figure 3c), Cyanobacteria (F_E*__V_ = 14.736, P_E*__V_ = 0.002) (Figure 3d), Spirochaetes (F_E*__V_ = 9.080, P_E*__V_ = 0.011) (Figure 3b) and Proteobacteria (F_E*__V_ = 26.336, P_E*__V_ < 0.001) (Figure 3g). The relative abundances of Cyanobacteria (Figure 3d) and Tenericutes (Figure 3c) in LZ+4 infection with *Epichloë* were significantly (*P* < 0.05) higher than that of EF, and reaches 92.57 and 125 times, respectively. However, the *Epichloë* (E) significantly (*P* < 0.05) inhibited the relative abundances of Spirochaetes (Figure 3b), Bacteroidetes (Figure 4e) and Proteobacteria (Figure 3g).

8 phyla bacteria community were detected in the LZ+4 and LQ+4 root infection with EF and EI. Proteobacteria, Actinobacteria and Bacteroidetes were the dominant group of bacteria in LZ+4 and LQ+4 roots (Figure 3 and Figure S1).

By analyzing the main groups of rhizosphere soil bacteria at the phylum level, we found that *Epichloë* (E) significantly affected the relative abundances of Nitrospirae (F_E_ = 6.408, *P_E_* = 0.030) (Figure 4c), Proteobacteria (F_E_ = 5.415, *P_E_* = 0.042) (Figure 4f), Actinobacteria (F_E_ = 6.620, *P_E_* = 0.028) (Figure 4d). The varieties (V) significantly affected the relative abundances of Planctomycetes (F_V_ = 6.797, *P_V_*= 0.026) (Figure 4b), Nitrospirae (F_V_ = 14.385, *P_V_* = 0.004) (Figure 4c), Actinobacteria (F_V_ = 41.911, *P_V_* < 0.001) (Figure 4d) and Acidobacteria (F_V_ = 5365, *P_V_* = 0.043) (Figure 4e). The *Epichloë* (E) and varieties (V) significantly affected the relative abundances of Actinobacteria (F_E*__V_ = 8.665, P_E*__V_ = 0.015) (Figure 4d). The relative abundances of Nitrospirae and Acidobacteria in LQ+4 infection with EI were significantly (*P*< 0.05) higher than that of EF, reaching 1.94 (Figure 4c) and 0.9 (Figure 4e) times. However, the *Epichloë* (E) significantly (*P* < 0.05) inhibited the relative abundances of Actinobacteria (Figure 4d) and Proteobacteria (Figure 4f).

11 phyla bacteria community were detected in the LZ+4 and LQ+4 rhizosphere soil infection with EF and EI. Proteobacteria, Acidobacteria and Bacteroidetes were the dominant group of bacteria in LZ+4 and LQ+4 (Figure 4 and Figure S1).

The bacterial composition of the rhizosphere soil was greater than that of the root at the phylum level (Figure 3, Figure 4 and Figure S1).

#### 3.5.2. Composition of Fungal Community in Root and Rhizosphere Soil

**Figure 5:**
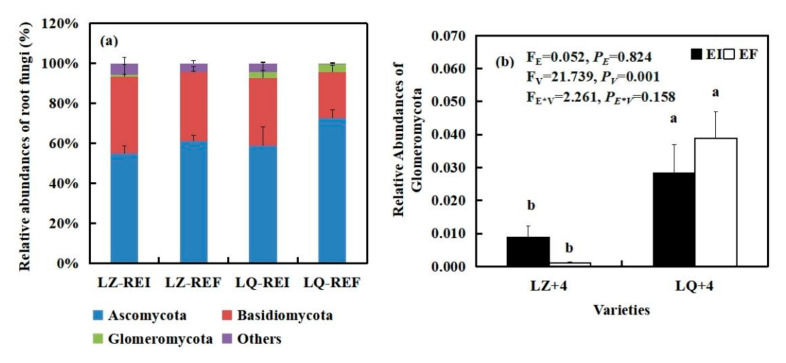
The relative abundance (at the phylum level) of root fungal community found in EI (*Epichloë*-infection) and EF (*Epichloë*-free) in LZ+4 and LQ+4. REI: endophyte-infection root, REF: endophyte-free root. (**a**) relative abundances of root fungi, (**b**) relative abundances of Glomeromycota. Results are presented as mean ± SE. Different lowercase letters indicate significant differences (*P* < 0.05) between the EI and EF barley (*n* = 4, *P* < 0.05). *P_V_*-values, *P_E_*-values, and *P_VE_* of the ANOVA indicate significant differences *P* < 0.05 (independent *t*-test) endophyte status (E), varieties (V), and their interaction (E*V), respectively.

By analyzing the main groups of root fungi at the phylum level, we found that varieties (V) significantly affected the relative abundances of Glomeromycota (F_V_ = 21.739, *P_V_* < 0.001). Glomeromycota was negatively affected by *Epichloë* infection in LQ+4 root and positively affected by *Epichloë* infection in LZ+4 root. The relative abundances of Glomeromycota of EI is 8 times that of EF in LZ+4 root (Figure 5b).

3 phyla fungal community were detected in the LZ+4 and LQ+4 root infection with EF and EI. Ascomycota and Basidiomycota were the dominant group in the root fungi community (Figure 5 and Figure S2).

**Figure 6:**
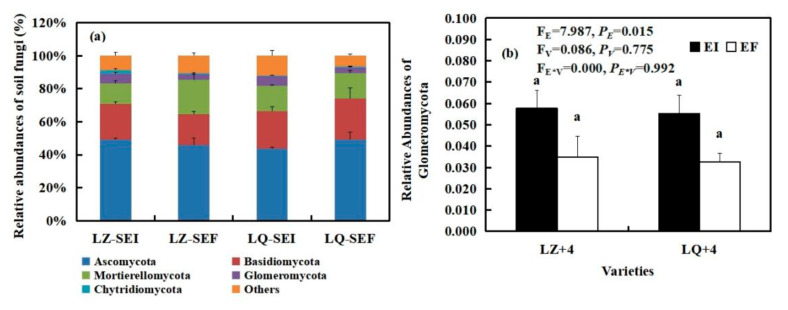
The relative abundance (at the phylum level) of rhizosphere soil fungal community found in EI (*Epichloë*-infection) and EF (*Epichloë*-free) in LZ+4 and LQ+4. SEI: endophyte-infection rhizosphere soil, SEF: endophyte-free rhizosphere soil. (**a**) relative abundances of soil fungi, (**b**) relative abundances of Glomeromycota. Results are presented as mean ± SE. Different lowercase letters indicate significant differences (*P* < 0.05) between the EI and EF barley (*n* = 4, *P* < 0.05). *P_V_*-values, *P_E_*-values, and *P_VE_* of the ANOVA indicate significant differences *P* < 0.05 (independent *t*-test) endophyte status (E), varieties (V), and their interaction (E*V), respectively.

By analyzing the main groups of rhizosphere soil fungi at the phylum level, we found that *Epichloë* (E) significantly affected the relative abundances of Glomeromycota (F_E_ = 7.986, *P_E_* = 0.015) (Figure 6b). The *Epichloë* (E) stimulated the relative abundances of Glomeromycota in LZ+4 and LQ+4 rhizosphere soil, EI is 1.65 and 1.69 times that of EF in LZ and LQ, respectively (Figure 6b).

5 phyla fungal community were detected in the LZ+4 and LQ+4 rhizosphere soil infection with EF and EI. Ascomycota, Basidiomycota and Mortierellomycota were the dominant group of rhizosphere soil fungi community in LZ+4 and LQ+4 infection with EF and EI (Figure 6 and Figure S2).

The fungal composition of the rhizosphere soil was greater than that of the root at the phylum level (Figure 5, Figure 6 and Figure S2).

### 3.6. Diversity and Richness of Bacterial and Fungal Community in Root and Rhizosphere Soil

**Figure 7:**
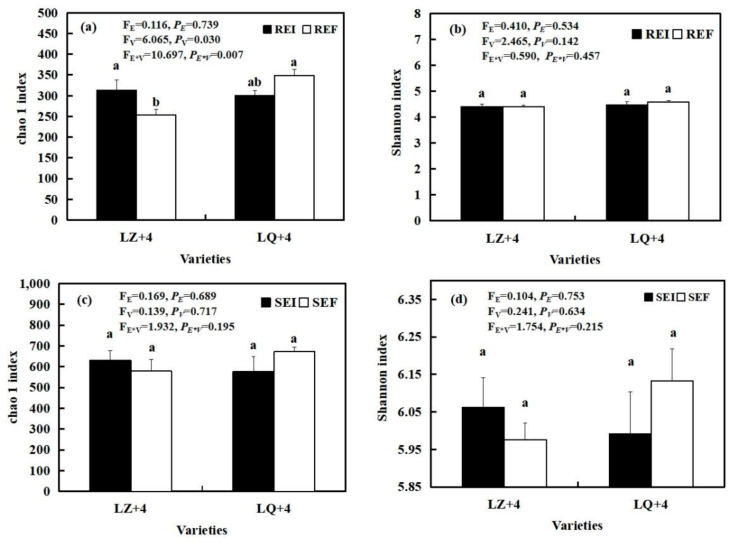
Bacterial diversity and richness in root-associated (**a**,**b**) and rhizosphere soil (**c**,**d**) in LZ+4 and LQ+4 infected with endophyte (*Epichloë*-infection; EI) and endophyte-free plants (*Epichloë*-free; EF). REI: endophyte-infected root, REF: endophyte-free, SEI: endophyte-infected rhizosphere soil, SEF: endophyte-free rhizosphere soil. Results are presented as mean ± SE. Different lowercase letters indicate significant differences (*P* < 0.05) between the EI and EF barley (*n* = 4, *P* < 0.05). *P_V_*-values, *P_E_*-values, and *P_VE_* of the ANOVA indicate significant differences *P* < 0.05 (independent *t*-test) endophyte status (E), varieties (V), and their interaction (E*V), respectively.

The interaction between varieties and *Epichloë* (F_E__*V_ = 10.697; P_E__*V_ = 0.007) (Figure 7a), and varieties (F_V_ = 6.065; *P_V_* = 0.03) (Figure 7a) had significant effects on the richness of the barley root-associated bacteria community, as summarized by the Chao 1 index. The richness of the EI barley associated bacteria community was significantly (*P* < 0.05) higher than that of EF in LZ+4 (Figure 7a), while the richness and diversity of the EF barley root and rhizosphere soil bacteria community was higher than that of EI with LQ+4 (Figure 7a–d).

**Figure 8:**
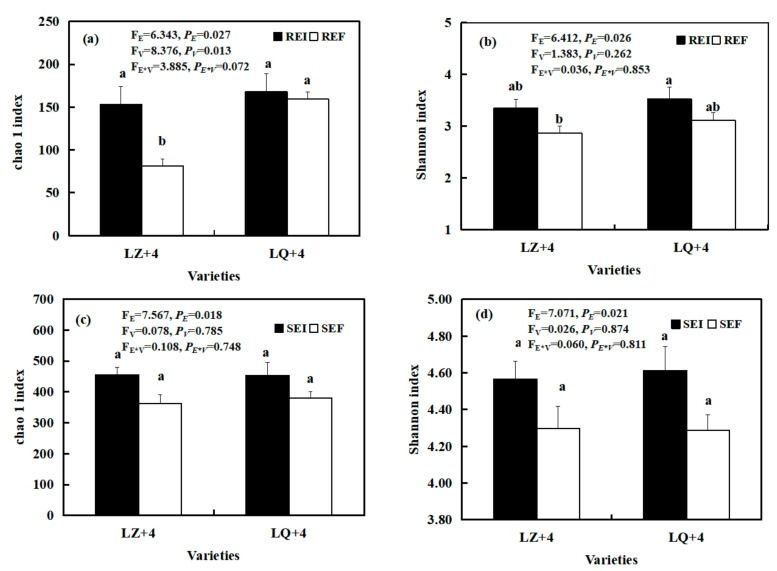
Fungi diversity and richness in root-associated (**a**,**b**) and rhizosphere soil (**c**,**d**) in LZ+4 and LQ+4 infected with endophyte (*Epichloë*-infection; EI) and endophyte-free plants (*Epichloë*-free; EF). REI: endophyte-infection root, REF: endophyte-free, SEI: endophyte-infection rhizosphere soil, SEF: endophyte-free rhizosphere soil. Results are presented as mean ± SE. Different lowercase letters indicate significant differences (*P* < 0.05) between the EI and EF barley (*n* = 4, *P* < 0.05). *P_V_*-values, *P_E_*-values, and *P_VE_* of the ANOVA indicate significant differences *P* < 0.05 (independent *t*-test) endophyte status (E), varieties (V), and their interaction (E*V), respectively.

The status of *Epichloë* had a significant effect on the diversity (F_E_ = 6.412, *P_E_* = 0.026; F_E_ = 7.071, *P_E_* = 0.021) and richness (F_E_ = 6.343, *P_E_* = 0.027; F_E_ = 7.567, *P_E_* = 0.018) of the root and rhizosphere soil fungal communities, as summarized by the Shannon and Chao1 indices, respectively (Figure 8a–d). The richness of the EI barley root fungal community was significantly (*P* < 0.01) higher than that of EF in LZ+4 (Figure 8a). The varieties had significant (F_V_ = 8.376, *P_V_* = 0.013) effects on the richness of the barley root fungal community (Figure 8a).

### 3.7. Beta Diversity Analysis

**Figure 9:**
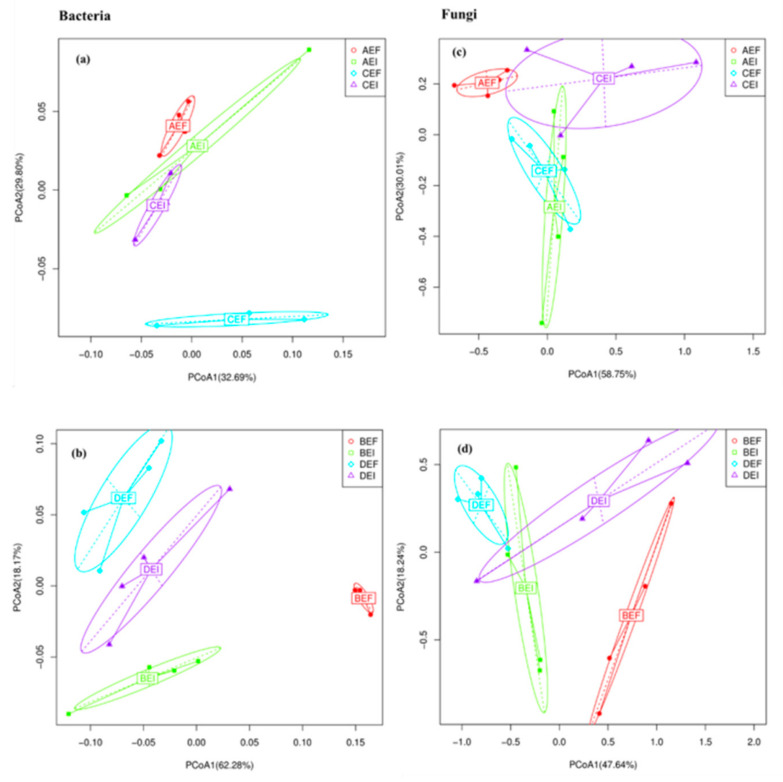
Principal coordinates analysis (PCoA) of LZ+4 and LQ+4 root-associated and rhizosphere soil bacterial (**a**,**b**) and fungal (**c**,**d**) communities at operational taxonomic units (OTUs) level based on the Bray–Curtis dissimilarities under different endophyte treatments. (*n* = 4; AEI: *Epichloë*-infection in LZ+4 root; AEF: *Epichloë*-free in LZ+4 root; BEI: *Epichloë*-infection LZ+4 rhizosphere soil; BEF: *Epichloë*-free in LZ+4 rhizosphere soil; CEI: *Epichloë*-infection LQ+4 root; CEF: *Epichloë*-free in LQ+4 root; DEI: *Epichloë*-infection LQ+4 rhizosphere soil; DEF: *Epichloë*-free in LQ+4 rhizosphere soil).

The PCoA results indicated the community composition of root and rhizosphere soil bacteria (a,b) and fungi (c,d) were significantly different between EI (*Epichloë*-infection) and EF (*Epichloë*-free), but were not significantly different in between varieties (Figure 9).

### 3.8. Relationships among Microbial Community and Environmental Factors with Peramine

Pearson correlation results indicated that the diversity and richness of the root fungi were significantly and positively (*P* < 0.05) associated with rhizosphere soil N/P and C/P, and significantly and negatively (*P* < 0.05) associated with rhizosphere soil P. The richness of rhizosphere soil fungi was significantly and negatively (*P* < 0.05) associated with soil P, but significantly and positively (*P* < 0.05) associated with peramine (Table S3).

**Figure 10:**
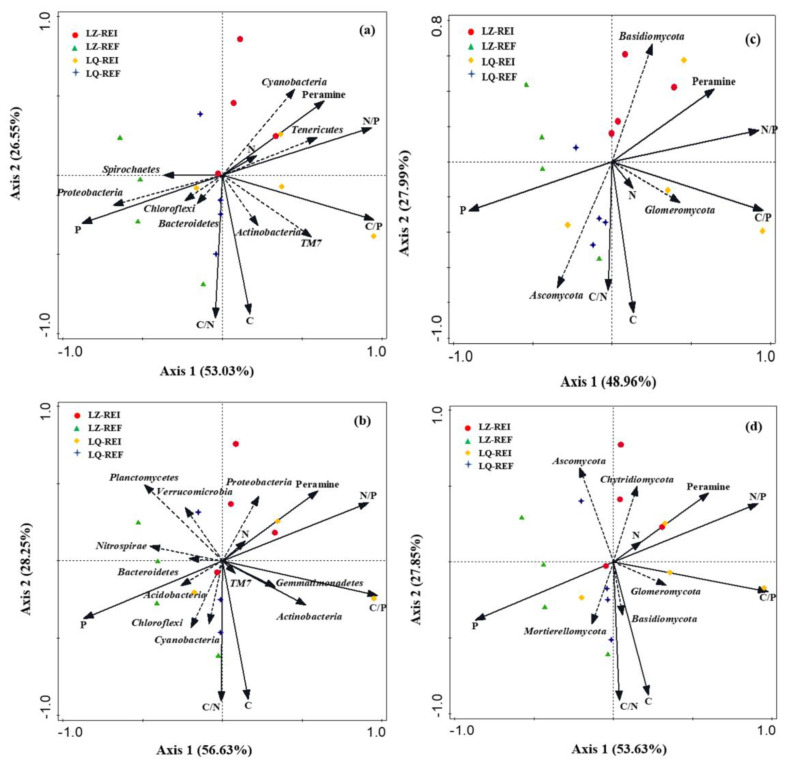
Redundancy analysis of relative abundance of root and rhizosphere soil bacteria (**a**,**b**) and fungi (**c**,**d**), soil properties, peramine, EI (*Epichloë*-infection) LZ+4 and LQ+4 and EF (*Epichloë*-free). Environmental factors include SOC (organic carbon), TP (total phosphorus), TN (total nitrogen), C/N (the OC and TN ratio), C/P (the OC and TP ratio) and N/P (the TN and TP ratio) (*n* = 4).

The first and second axis of RDA among the root bacteria community and soil properties and peramine explained 53.03% and 26.55% of the variance (Figure 10a), respectively. The content of peramine was positively related to the N, N/P, Tenericutes and Cyanobacteria. Additionally, the first and second axis of RDA among the rhizosphere soil bacteria community and soil properties and peramine explained 56.63% and 28.25% of the variance (Figure 10b), respectively. The content peramine were positively related to the N, N/P, C/N, and Proteobacteria.

The first and second axis of RDA among the root fungi community and soil properties and peramine explained 48.96% and 27.99% of the variance (Figure 10c), respectively. The content peramine was positively related to the N/P and Basidiomycota. Additionally, the first and second axis of RDA among the rhizosphere soil fungi community and soil properties and peramine explained 53.63% and 27.85% of the variance (Figure 10d), respectively. The content peramine was positively related to the N, C/P, N/P and Chytridiomycota.

## 4. Discussion

### 4.1. Endophyte Status and Barley Cultivar Affect Barley Growth Parameters, Toxicity and Quality

Overall, our results support the first hypothesis that plant growth parameters are significantly influenced by the fungal endophyte. Our results are consistent with those from several studies reporting *Epichloë*-associated increases in biomass production in E+ plants relative to E− plants [17]. Conversely, “Monad” wheat (*Triticum aestivum*) infected with an *Epichloë* strain sourced from *Elymus dahuricus* subspecies excelsus—strain AR3060 were stunted or dwarfed [47]. Moreover, Kenyon [48] observed no differences in annual dry matter (DM) production for E− and E+ tall fescue. This indicates that *Epichloë* is triggering some form of host response, and this response varies according to the genotype of the individual host plant. The reason for this lack of difference in barley growth parameters is unclear but may be related to vegetative growth features particular to barley.

Importantly, from the perspective of strain deployment, it is necessary to analyze for alkaloid production in plants to confirm their efficacy. By inoculating perennial ryegrass varieties, with a few novel strains with desirable metabolite profiles, unique cultivars have been created for commercial production. For example, the commercial novel endophytes NEA2, NEA3 and NEA6 produce both ergovaline and peramine, but not lolitrem B [49]. By contrast, the novel endophyte AR1 [50] produce peramine but does fails to produce lolitrem B and ergovaline. The *E. bromicola* LZ+4 strain produces peramine, chanoclavine I, D-lysergic acid, and ergovaline but fails to produce ergonovine [34]. Peramine was only detected in EI plants of LZ+4 and LQ+4. This supports our first hypothesis. The peramine concentrations can be as high as 0.1 μg/g and 0.08 μg/g in LZ+4 and LQ+4, respectively. Previous studies have indicated that the peramine deterred the feeding of both adults and larvae of the gramineous herbivore, Argentine stem weevil (*Listronotus bonariensis*), at 0.1 μg/g and 10 μg/g, respectively [51]. In this study, the peramine content of LZ+4 reached the insecticidal threshold. Chen [52] showed that endophyte infection rates in seeds increased with the postponement of the sowing year, which is opposite with our observations of endophyte infection rates in seed and tillers that decreased over time. This is because one is natural breeding, and the other is artificial inoculation. Simpson found that some grasses successfully inoculated with endophyte lose infection during seed storage. In the process of progeny growth, some tillers could not successfully transmit the endophyte, resulting in loss of the endophyte in those tillers and seeds [47]. Our study confirmed that the infection rates in seeds and tillers were inconsistent, Additionally, the infection rate of the fourth generation plants reached 47–76%. Therefore, we need further research how to improve the carrier rate and alkaloid content of new symbionts. In summary, An understanding of the respective biology and genetics combined with experimental investigation is likely to yield positive results in the attempt to produce agriculturally useful synthetic novel symbioses.

### 4.2. Endophyte Status and Barley Cultivar Affects the Relative Abundances and Diversity of Root-Associated and Rhizosphere Microbial Communities

To our knowledge, this is the first study to quantify the effects of the aboveground *E. bromicola* endophyte on root-associated and rhizosphere soil microbial community of *E. bromicola*-barley germplasm using sequencing techniques. The *Epichloë* of tall fescue increased the rhizosphere soil bacteria diversity [53]. Ju [54] found that *Epichloë gansuensis* enhanced the diversity and richness of rhizosphere soil bacteria community with *A. inebrians*. In contrast, Mahmud [10] found that the *Epichloë*-infection tall fescue soil showed a lower bacteria community diversity at the genus level compared to the fungal community. Our results support the second hypothesis that *E.* *bromicola*-barley symbiosis increased the diversity and richness of the root-associated and rhizosphere soil fungal community in LZ+4 and LQ+4, but had no significant effect on bacteria community. Different effects on plants could result from the following: (1) plant inoculation with sexual fungal native or non-native endophytes [55] could alter host grass nutritional requirements [56], and this may indirectly affect root-associated and rhizosphere microbial communities; (2) different *Epichloë* can produce different primary or secondary metabolites (e.g., alkaloids) [57] or root exudates [17] that could directly affect the root-associated and rhizosphere microbial communities; (3) apart from the endophyte species, plant genotypes and plant species determine plant root-associated and rhizosphere microbial communities [58]. Generally speaking, the present study confirmed the second hypothesis that *Epichloë* had an effect on soil and root microbial community diversity.

Our results support the second hypothesis that *Epichloë* had an effect on soil and root microbial community composition. Proteobacteria and Acidobacteria are copiotroph and oligotroph microbial taxa of rhizosphere bacterial communities. Acidobacteria offers efficient carbon and nitrogen cycling from soil organic matter that can consequently be used as a readily available nutrient source for plants [59]. Proteobacteria can release nutrients from complexes of organo-mineral that facilitate plant growth [60]. The distinction between oligotrophs and copiotrophs can be further studied to expose the resource use characteristics of the community. Copiotrophs favor high carbon environments, while the oligotrophs outcompete copiotrophs in low resource environments [61]. The ratio of Proteobacteria to Acidobacteria (P/A) can be used as a general indicator of soil nutrient status; low P/A ratio indicates poor soil environment, and high P/A ratio indicates nutrient richness [62]. In our study, the percent abundance ratio of Proteobacteria/Acidobacteria (P/A) was higher in E+ LZ+4 rhizosphere soil (2.05) compared to E− (1.84). These results indicate that E+ have higher soil nutrient status compared to E−, and E− LZ+4 performs poorer in overall plant fitness and persistence. Contrary conclusions were made by Mahmud [10], who also found that Proteobacteria/Acidobacteria (P/A) was lower in E+ tall fescue rhizosphere soil (1.66) compared to E- tall fescue rhizosphere soil (2.57), as our data suggest that E− have higher P/A compared to E+ in LQ+4. These results indicate that E− have higher soil nutrient status compared to E+, possibly due to the lower percent abundance of the Proteobacteria phylum in E+ vs. E−. Glomeromycota are arbuscular mycorrhizal fungi (AMF) and are known to play diverse roles in soils that can impact other organisms and soil function, such as carbon sequestration [63]. AMF are symbiotic with 80% of terrestrial plants [64]. Previous studies indicated that *Epichloë coenophiala* strains in tall fescue stimulated (rather than reduced) the relative abundance of Glomeromycota phylum [65] in soil. Another study showed that Glomeromycota are also present in the root system, and *E. gansusensis* increased their relative abundance [66], as our data suggest, which may be the reason for the increase in soil carbon storage. Antunes [20] found the presence of *Epichloë bromicola* had no effect on Glomus mosseae and Glomus etunicatum in Leymus chinensis. The possible reasons for these gaps are ryegrass cultivar and endophyte strain [67]. The present results contrast with these findings in that *E. bromicola* strains improved Glomeromycota relative abundance in all samples, except for LQ+4 roots. *E. bromicola* strains improved Ascomycota relative abundance in all samples, except for LZ+4 soil. Previous studies have shown the combined presence of Ascomycota, Basidiomycota, and Glomeromycota in E + tall fescue soil suggests that the presence of endophyte in tall fescue affects the rhizosphere fungal community structure, possibly through a combination of factors, which may further contribute to the promotion of plant growth [18,68], as our data suggest that *E. bromicola* strains also improved Ascomycota relative abundance in all samples, except for LZ+4 soil. Thus, the presence of a complex fungal assemblage at genus level in E + barley soil suggests that root excreted substances from E + barley into the soil may have enhanced the mobilization or recruitment of beneficial rhizosphere fungal communities, and in turn, these different soil fungal communities possibly could provide greater fitness and resilience to the plant [69].

The present study showed that the diversity and richness of the rhizosphere soil fungal and bacterial communities are significantly higher than those within the root-associated fungal and bacterial community, as observed by other studies which showed that the diversity and richness of the rhizosphere soil AM fungal community are significantly higher than those within the root-associated AM fungal community in *Achnatherum inebrians* [66], grassland [70] and maize [71]. Ju [54] found that the diversity of bacterial community in rhizosphere soil had a higher diversity than in the roots. Another study found that the diversity of these bacterial communities decreased from soil to roots in wheat [72] and rice [73]. This may be because soil nutrient status plays a central role in impacting soil bacterial and fungal communities. The root system is the link between plant and soil. Plant roots release secondary metabolites into the soil, and this serves as an energy source for rhizosphere soil microbial populations [74]. Bacterial communities tightly bound to the root are simplest [72]. In contrast to these findings, it was found that bacterial diversity and richness in the root system was significantly higher than in the soil in *Ammophila breviligulata*, a grass that thrives in sand dunes. This may be because root exudates provide root bacteria with much-needed resources in sandy soil of the dune ecosystem [58].

### 4.3. Relationship among the Diversity in Root and Rhizosphere Soil Bacterial and Fungal Communities, with Soil Properties and Peramine

Previous studies have shown that the diversity and composition of microbial communities associated with plant roots and rhizosphere soil are affected by different *Epichloë* strains including *Epichloë coenophiala* [65], *Neotyphodium lolii* [67], *E. gansuensis* [54], *Epichloë bromicola* [20] and environmental factors including nitrogen [75], phosphorus [10], and pH [76]. These factors normally lead to changes in the physical and chemical properties of rhizosphere soil and soil nutrient levels, which are closely related to the diversity of rhizosphere soil microbial communities [77]. The availability of inorganic nitrogen regulates the relative diversity of bacteria and archaea of soil microbial communities; in return, bacteria are involved in the whole process of inorganic nitrogen cycling [78]. Previous studies have shown that high levels of N addition altered the composition of the forest soil bacterial community and decreased soil bacterial diversity [79]. Moreover, the effects of P inputs on the soil bacterial community structure in the field was lower than that of N or K inputs [80]. Ju [54] also highlighted that soil N and AP were closely correlated with rhizosphere soil bacterial diversity of *A. inebrians*. Our study also found that N was significant and had positive effect on the Cyanobacteria and Tenericutes in root and Proteobacteria in rhizosphere soil. This plant-available P in the rhizosphere is likely to contribute to soil bacterial and fungal growth [10]. The presence or absence of the *Epichloë*, and the diversity and richness of the fungal communities in rhizosphere soil and root, were closely related to the availability of soil P [66]. Available P has a significant effect on the diversity and richness of the AM fungal community in *Pennisetum centrasiaticum* and *Kobresia* sp. [81]. Additionally, Glomeromycota abundance in the soil fungal community was significantly positively correlated to soil NN [82]. Our study found that the diversity and richness of the fungi community in root and the richness of fungi in rhizosphere soil were suppressed by P, and N promoted the relative abundance of Glomeromycota in root and rhizosphere soil.

Three endophyte strains, AR542E+, CTE+ and AR584E+, had similar effects on the soil fungal community [53], but they have different capacities for producing alkaloids [83]. This suggests that *Epichloë*-produced alkaloids were not responsible for the observed changes in fungal communities [20]. The interaction between endophytes and soil fungi is not related to alkaloid production, which is consistent with the fact that alkaloids are rarely measured in roots [19]. What is more, the alkaloids are found only within the plant and have not been identified in exudates released from plant roots into the rhizosphere [18]. However, the alkaloids produced by foliar *Epichloë* inhibit the spore germination of *G. intraradices* compared to that of EF plants in soil [18]. Moreover, studies have detected the presence of endophyte-produced loline alkaloids in the roots of tall fescue [84]. Subsequently, alkaloids were found in surface soils in pastures dominated by CTE+ tall fescue [57]. Peramine alkaloids are present in aboveground *Epichloë bromicola*-barley tissues in our study. The present study confirmed the third hypothesis that peramine was positively correlated with fungal Chao richness of the rhizosphere soil, and the greater relative abundance of Ascomycota in rhizosphere soil and Basidiomycota in root were positively with peramine. Similar conclusions were made by Guo [18], who found that the greater relative abundance of Ascomycota and Basidiomycota of tall fescue in rhizosphere soil, respectively, suggests that alkaloids (loline or peramine) produced by endophyte affects the rhizosphere fungal community structure. It may be due to endophyte-induced changes in barley physiology, possibly increasing soil properties [54,66] to alter peramine providing more nutrients to promote the microbial community. Our study also validated that peramine was highly positively correlated with soil N, and peramine alkaloids and soil N had the same effects on rhizosphere soil and root microbial communities at the phylum level. However, no studies were conducted to determine peramine presence in the roots and rhizosphere soil associated with *E. bromicola*-barley combination. Thus, further research is needed to verify that foliar *E. bromicola* affected the below-ground microbial communities through alkaloids of the *E. bromicola*-barley combination.

## 5. Conclusions

This is the first study on inoculation of barley with endophytes in recent years. In this study, we investigated the possible roles of interaction between *Epichloë* infection and barley varieties on the above-ground and below-ground parts of barley. This study revealed that inoculation with *E. bromicola* promotes barley growth. Moreover, *Epichloë*-barley symbionts produced peramine. In addition, the presence of *E. bromicola* in barley significantly increased the Shannon diversity and Chao richness of fungi in the root and rhizosphere soil. Moreover, the richness of *E. bromicola* root-associated fungi communities were intimately associated with soil properties of available P and peramine. The possible mechanisms by which *Epichloë* infection enhanced the growth of barley include regulating the alkaloid peramine synthesis and changing soil fertility by altering root and rhizosphere soil microbial diversity. Our results have both theoretical and practical significance. Collectively, based on the knowledge of *E. bromicola*, we provide a theoretical and practical basis for *E. bromicola*-infection barley to improve their performance.

## Data Availability

The datasets generated (soil bacteria: PRJNA782467, root bacteria: PRJNA782458, siol fungi: PRJNA783082, root fungi: PRJNA783073) for this study can be found in the Sequence Read Archive (SRA) (https://www.ncbi.nlm.nih.gov/sra/?term=PRJNA590316, accessed on 25 November 2021).

## Supplementary Materials

The following supporting information can be downloaded at: https://www.mdpi.com/article/10.3390/jof8020172/s1, Figure S1: The relative abundance of bacteria operational taxonomic units (OTUs) (a,b) in all root samples, OTUs (c,d) in all rhizosphere samples in EI (*Epichloë*-infection) and EF (*Epichloë*-free) LZ+4 and LQ+4. (*n* = 4, a and c: LZ+4; b and d: LQ+4).; Figure S2: The relative abundance of fungal operational taxonomic units (OTUs) (a,b) in all root samples, OTUs (c,d) in all rhizosphere samples in LZ+4 and LQ+4 infected with in EI (*Epichloë*-infection) and EF (*Epichloë*-free). (*n* = 4, a and c: LZ+4; b and d: LQ+4). Table S1: Inoculation rate changes of *E. bromicola*-barley new symbionts. LZ: *E. bromicola* were not successfully inoculated into *H*. *vulgare* cv. Yangsimai No. 1, LQ: *E. bromicola* were not successfully inoculated into H. vulgare var. nudum cv. Chaiqing No. 1. LZ+4 and LQ+4: Fourth generation of LZ and LQ inoculation with Epichloë harvested in 2020. Table S2: Rhizosphere soil properties associated with different kinds of barley under varieties (V) and endophyte (E) treatments, and results of two-way analysis of variance (two-way ANOVA) for the S and E on rhizosphere soil properties. E*V: interaction of *E. bromicola* and species. SOC: organic carbon, TP: total phosphorus, TN: total nitrogen, C/N: the OC and TN ratio, C/P: the OC and TP ratio, N/P: the TN and TP ratio. * indicates *P* < 0.05. Table S3: Pearson correlations of alpha diversity in root and rhizosphere soil bacteria and fungi community to soil properties and peramine. SOC: organic carbon, TP: total phosphorus, TN: total nitrogen, C/N: the OC and TN ratio, C/P: the OC and TP ratio, N/P: the TN and TP ratio. * indicates *P* < 0.05, ** indicates *P* < 0.01.
