# Supplementary material for: Inoculation of Barley (Hordeum vulgare) with the Endophyte Epichloë bromicola Affects Plant Growth, and the Microbial Community in Roots and Rhizosphere Soil
Source: J Fungi (Basel). 2022 Feb 10;8(2):172. doi: 10.3390/jof8020172 (PMC8876963; doi:10.3390/jof8020172)
Supplement: Supplementary file 1 [file jof-08-00172-s001.zip › Supp-Tables.pdf]

Table S1 Inoculation rate changes of *E. bromicola*-barley new symbionts. LZ: *E.bromicola* were not successfully inoculated into *Hordeum vulgare* cv. Yangsimai No. 1, LQ: *E.bromicola* were not successfully inoculated into *H. vulgare* var. nudum cv. Chaiqing No. 1. LZ+4 and LQ +4: Fourth generation of LZ and LQ inoculation with *Epichloë* that harvested in 2020.

Association	Plant No. tested	Plant No. inocolation	Inocolation rate	Seed No. tested	seed No. inocolation	Inocolation rate
LZ	20	0	0	50	0	0
LZ+4	50	38	76	100	56	56
LQ	20	0	0	50	0	0
LQ+4	50	34	68	100	47	47

Table S2 Rhizosphere soil properties associated with different kinds of barley under varieties (V) and endophyte (E) treatments, and results of two-way analysis of variance (two-way ANOVA) for the S and E on rhizosphere soil properties. E*V: interaction of *E. bromicola* and species. SOC:Organic Carbon, TP:Total Phosphorus, TN:Total nitrogen, C/N:The OC and TN ratio, C/P:the OC and TP ratio, N/P:The TN and TP ratio. * indicates $P < 0.05$.

Varieties	Tretments	TN (g/kg)		TP (g/kg)		SOC (g/kg)		N/P (%)		C/N (%)		C/P (%)	
LZ+4	EI	1.077±0.008		0.520±0.055		6.440±0.477		2.080±0.110		6.013±0.462		12.453±0.758	
	EF	1.067±0.007		0.663±0.026		7.197±0.468		1.610±0.049		6.747±0.439		10.883±0.962	
LQ+4	EI	1.153±0.020		0.533±0.070		7.343±0.348		2.237±0.292		6.377±0.214		14.267±1.964	
	EF	1.12±0.031		0.607±0.003		7.237±0.326		1.850±0.055		6.473±0.460		11.927±0.510	
		F	*P*	*F*	*P*	*F*	*P*	*F*	*P*	*F*	*P*	*F*	*P*
	E	2.020	0.193	0.218	0.653	1.320	0.284	1.526	0.252	0.012	0.915	1.453	0.263
	S	0.224	0.648	5.459	**0.048***	0.627	0.451	7.120	**0.028***	1.038	0.338	2.722	0.138
	E*S	0.065	0.805	0.570	0.472	1.106	0.324	0.067	0.802	0.611	0.457	0.106	0.754

Table S3 Pearson correlations of alpha diversity in root and rhizosphere soil bacteria and fungi community to soil properties and peramine. SOC:Organic Carbon, TP:Total Phosphorus, TN:Total nitrogen, C/N:The OC and TN ratio, C/P:the OC and TP ratio, N/P:The TN and TP ratio. * indicates $P < 0.05$, ** indicates $P < 0.01$.

Soil properties	Root								rhizosphere soil							
	Fungi				bacteria				Fungi				bacteria			
	Shannon		Chao1		Shannon		Chao1		Shannon		Chao1		Shannon		Chao1	
	F	*P*	F	*P*	F	*P*	F	*P*	F	*P*	F	*P*	F	*P*	F	*P*
N	0.086	0.751	0.067	0.806	-0.033	0.904	0.035	0.897	-0.028	0.917	-0.094	0.728	0.018	0.952	0.036	0.903
P	-0.724**	0.002	-0.623**	0.01	0.319	0.228	-0.129	0.635	-0.427	0.099	-0.532*	0.034	-0.1	0.734	-0.067	0.82
C	-0.122	0.654	0.115	0.671	0.409	0.116	0.24	0.37	0.194	0.472	-0.037	0.893	0.126	0.667	0.113	0.7
N/P	0.669**	0.005	0.603*	0.013	0.287	0.282	0.14	0.605	0.388	0.137	0.428	0.098	0.17	0.56	0.142	0.627
C/N	-0.109	0.687	0	0.999	0.426	0.1	0.124	0.648	0.202	0.454	0.106	0.695	0.11	0.708	0.118	0.688
C/P	0.53*	0.035	0.602*	0.014	-0.034	0.901	0.243	0.364	0.466	0.069	0.395	0.13	0.29	0.314	0.255	0.379
Peramine	0.334	0.206	0.419	0.107	-0.055	0.841	0.261	0.329	0.392	0.133	0.516*	0.041	0.172	0.558	0.198	0.498
