# Supplementary figures and images for: Inoculation of Barley (Hordeum vulgare) with the Endophyte Epichloë bromicola Affects Plant Growth, and the Microbial Community in Roots and Rhizosphere Soil
Source: J Fungi (Basel). 2022 Feb 10;8(2):172. doi: 10.3390/jof8020172 (PMC8876963; doi:10.3390/jof8020172)

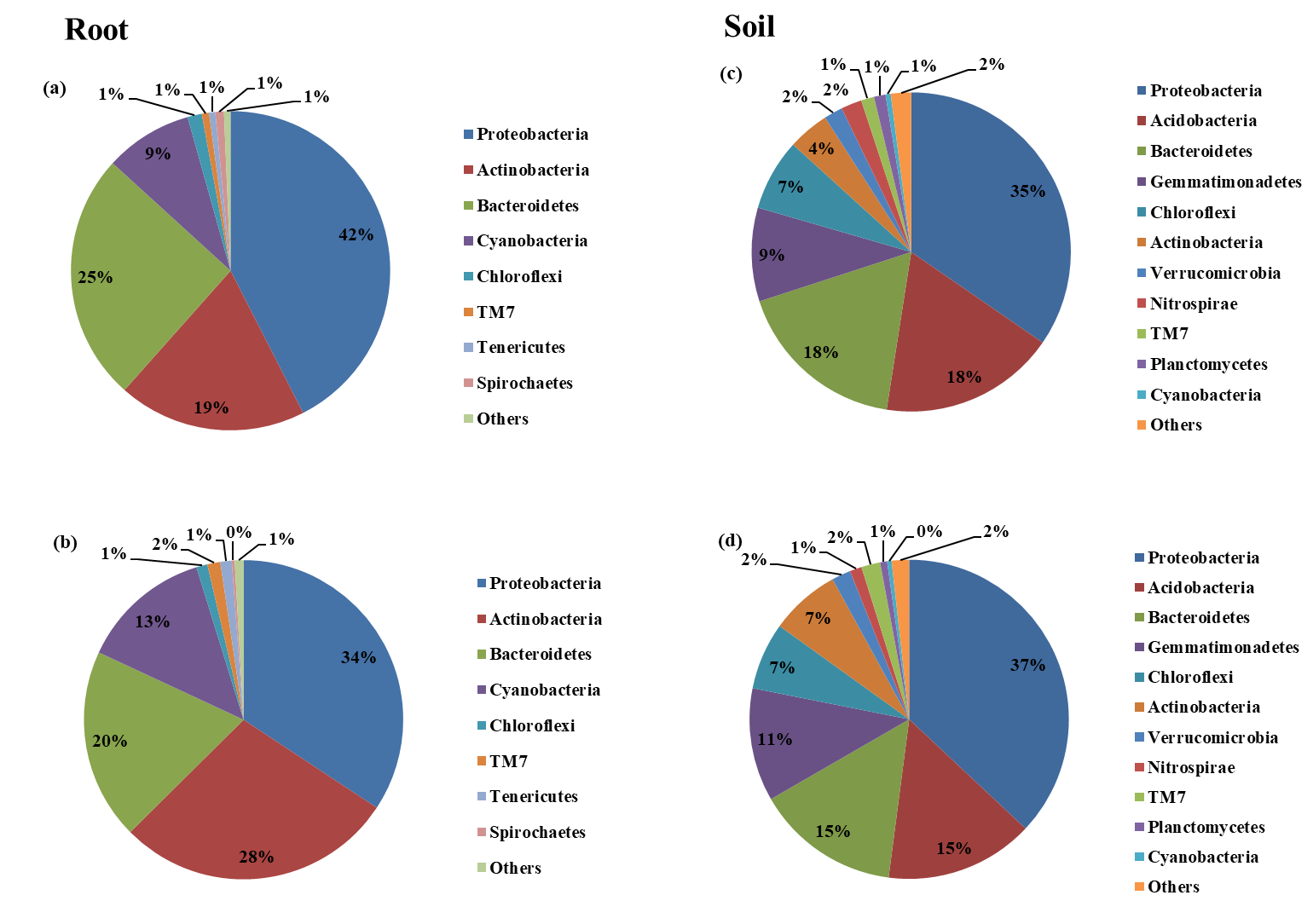

Supplement: Supplementary file 1 [file jof-08-00172-s001.zip › Figure S1.png]

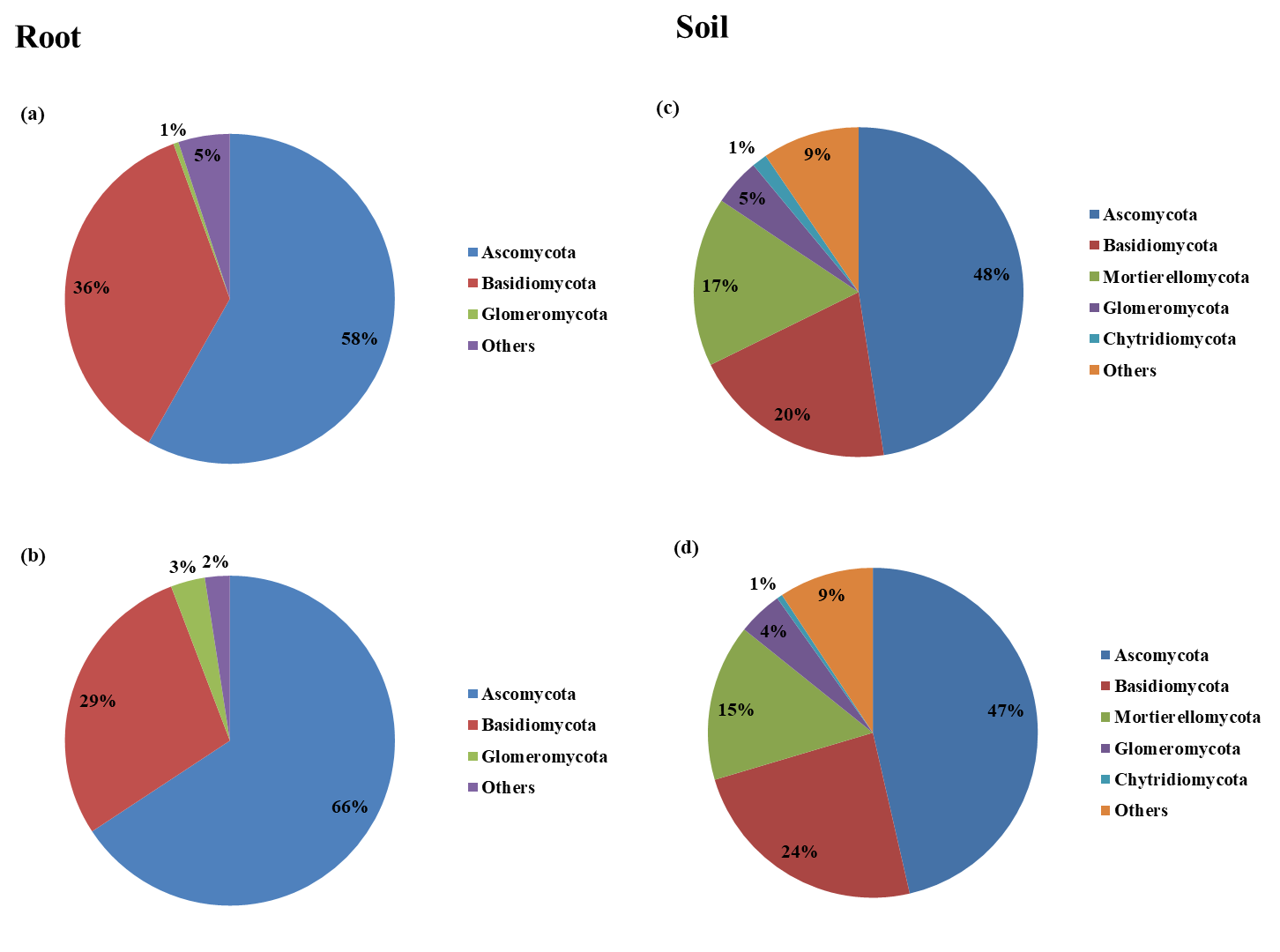

Supplement: Supplementary file 1 [file jof-08-00172-s001.zip › Figure S2.png]
